# A long tail of truth and beauty: A zigzag pattern of feather formation determines the symmetry, complexity, and beauty of the peacock’s tail

**DOI:** 10.12688/f1000research.149948.3

**Published:** 2025-05-06

**Authors:** Rama Singh, Santosh Jagadeeshan

**Affiliations:** 1 Biology, McMaster University, Hamilton, Ontario, L8S 4A9, Canada

**Keywords:** peacock’s tail, sexual selection, mate choice, female choice theory, eyespots, complexity, symmetry

## Abstract

**Background:**

Darwin assumed that the peacock’s long train was maladaptive and was the indirect effect of selection by female mate choice based on the train’s beauty. While a relationship between the feathers elaborate features and mating success has been shown in some studies, what features of the train females are attracted to remains controversial.

**Methods:**

We used museum specimens to examine the anatomical plan underlying feather development responsible for the train’s symmetry. We developed a model based on an alternate arrangement of primordial feather buds during development and locations of concentric circles of eyespot distribution using the pattern on the train as a template.

**Results:**

We observed a zigzag pattern of feather follicles that determined both the number and the arrangement of eyespots on the train. Our model explains the bilateral symmetry of train feathers, the hexagonal arrangement of eyespots on the train, and the concentric color rings of the eyespots. While the zigzag pattern explains the symmetry, complexity, and (structural) beauty of the peacock’s train, it also precludes variation in eyespot number except by annual addition of new feathers as a function of age.

**Conclusions:**

We propose a multimodal model of mate choice which holds that (1) eyespot number and feather length are developmentally correlated and females see them not as separate traits but as one complex trait combining both, (2) females may not always choose males with the largest number of eyespots, as old males may lack vigor, and (3) females may choose mates on the basis of train size, vigor, and beauty. The maladaptation of the long tail is a byproduct of the adaptation of the tall train. Who would have thought that zigzag arrangement, the densest form of spherical packing, when applied to the living world would produce such profound effects on phenotypic diversity.

*Beauty is truth, truth beauty.*

        Keats

## Introduction

The Indian blue peacock’s, *Pavo cristatus,* elaborate and long tail has long represented a paradigmatic case for the theory of sexual selection by female choice. However, even after 150 years, the problem of the evolution of the peacock’s long tail remains unsolved—we still do not know the basis (target) of female choice in peafowl. Darwin (1859) recognized the peacock’s long tail as a problem for his theory of evolution by natural selection as he conjectured it was too long to be of adaptive use to the animal; therefore, it was maladaptive. There are three aspects of the peacock’s tail, length, structural complexity, and “beauty,” that need to be considered together. By beauty here we are concerned with the structural arrangement and the color of the eyespots, which adds to the overall beauty of the peacock’s tail. Darwin chose to focus on the visual beauty of the tail and supplied an explanation through sexual selection: that females may prefer to mate with males who possess more beautiful and elaborate tails ( Darwin, 1871). It was thought that this reproductive advantage enjoyed by males with more elaborate tails would compensate for any loss of male fitness such as reduced survivorship due to predation. This explanation sets the stage for research to focus on elucidating how females assess “beauty” or attractive traits in their choice of mates ( Andersson, 1994).

Two key requisites for evolutionary theory, be it via natural or sexual selection, are variation and heritability. If the peacock’s train is a target of female choice, then there must be genetic or phenotypic variation in female preference that would directly or indirectly depend on variation in the peacock’s train morphology. Although female choice is widely assumed to be responsible for the evolution of the peacock’s tail, research on this matter has produced mixed results to date. While several researchers have shown a relationship between train features and mating success ( Petrie et al., 1991; Petrie and Halliday, 1994; Yasmin and Yahya, 1996; Loyau et al., 2005; Dakin and Montgomerie, 2011, 2013; Harikrishnan et al., 2010) controversy remains. For example, a comprehensive 7-year re-evaluation by Takahashi et al. (2008) found no evidence of train morphology (either train length or eyespot number) influencing the number of copulations a male achieves. More importantly, this re-evaluation uncovered little variation in train morphology across populations, thereby calling into question the idea that the peacock’s train is a target of female choice (however, see Loyau et al., 2008). Dakin and Montgomerie (2011) found that males over the age of four generally produce between 165 and 170 eyespots before the onset of the mating season. They found little variation within and between populations in terms of eyespot number, and rather intuitively suggested that this lack of variation in train morphology possibly reflects developmental constraints. There may be variation in eyespot number between growing males ( Manning, 1989) but the number in adult males appears invariant ( Manning, 1989; Takahashi et al., 2008; Dakin and Montgomerie, 2011).

To explain the remarkable symmetry of the structural arrangement and iridescence of the train and to understand the nature of variation in eyespot number we investigated the anatomical development plan of peacock train morphology. We uncovered developmental and anatomical evidence demonstrating that the nature of bilateral symmetry in the development of the peacock’s train feathers would preclude genetic variation in the number of individual eyespots. In this report we show, first, that a simple zigzag pattern of feather formation giving rise to hexagonal arrangement of eyespots uniquely determines the symmetry, complexity, and beauty of eyespots in the peacock’s tail. Second, we show that eyespot feathers originate in alternate, zigzagging rows of 10/11 annually, making the total number of eyespots an intrinsically determined trait. Third, we argue that both feather number ( Manning, 1989) and feather growth are asymptotic functions of age that may explain the contradictory results reported between different studies ( Loyau et al., 2008; Takahashi et al., 2008). We discuss the implications of these results, especially the lack of genetic variation in eyespot number, for sexual selection theories. We propose a multimodal model of female choice based on male’s train size, vigor, and beauty to understand how sexual selection operates ( Mitoyen et al., 2019) and how females choose mates. We propose that many conflicting results can be explained by assuming that females do not base their mate choice on eyespot number or train size alone but on a complex trait combining both.

## Methods

### Peacock specimens

The data presented in this report are based on observations of the tail structures of museum specimens kept at the Royal Ontario Museum, Toronto, Canada, and the American Museum of Natural History, New York, USA. Only specimens in good condition were included in this study. A total of 21 samples of blue fowl ( *P. cristatus*), including two albinos and two hybrids were examined. The wild blue fowl samples originated from India (N = 3), Sri Lanka (N = 2), Kenya (N = 1), or were captive samples originating from the United States (N = 4 blue, N = 2 albino). The remaining 11 samples were of unknown origin and included two hybrids. We also had access to seven green fowl ( *P. muticus*) samples at the Natural History Museum, which originated from Malaysia (N = 4), Bangkok (N = 1), or were of unknown origin (N = 2). Wherever dates were noted, most of the field samples were collected during the early 1900s. Conversely, the captive samples were collected as recently as 1942.

### Data collection

In this study, we use *tail* and *train* interchangeably, as appropriate- former to signify the long horizontal tail, the latter in expanded vertical position. We counted the number of eyespots, eyespot feathers, and fishtail feathers displayed by the included specimens ( Figure 3). In cases where an eyespot was missing due to damage, we counted it as if it were present to estimate the total number of eyespots. Very few of the samples had a uropygium that was in good condition. Of the museum specimens six had intact uropygium in *P. cristatus* and one in *P. muticus.* Wherever we were certain that the integrity of the sample had not been compromised, we counted the rows of feather imprints on the uropygium ( Figures 2B, 4). Dryad: A long tail of truth and beauty: The developmental basis of complexity, symmetry, and beauty in the evolution of the peacock’s tail. Dryad My Dataset: doi: 10.5061/dryad.1g1jwstwg.

### Graphical simulation of train expansion

**Figure 1.**
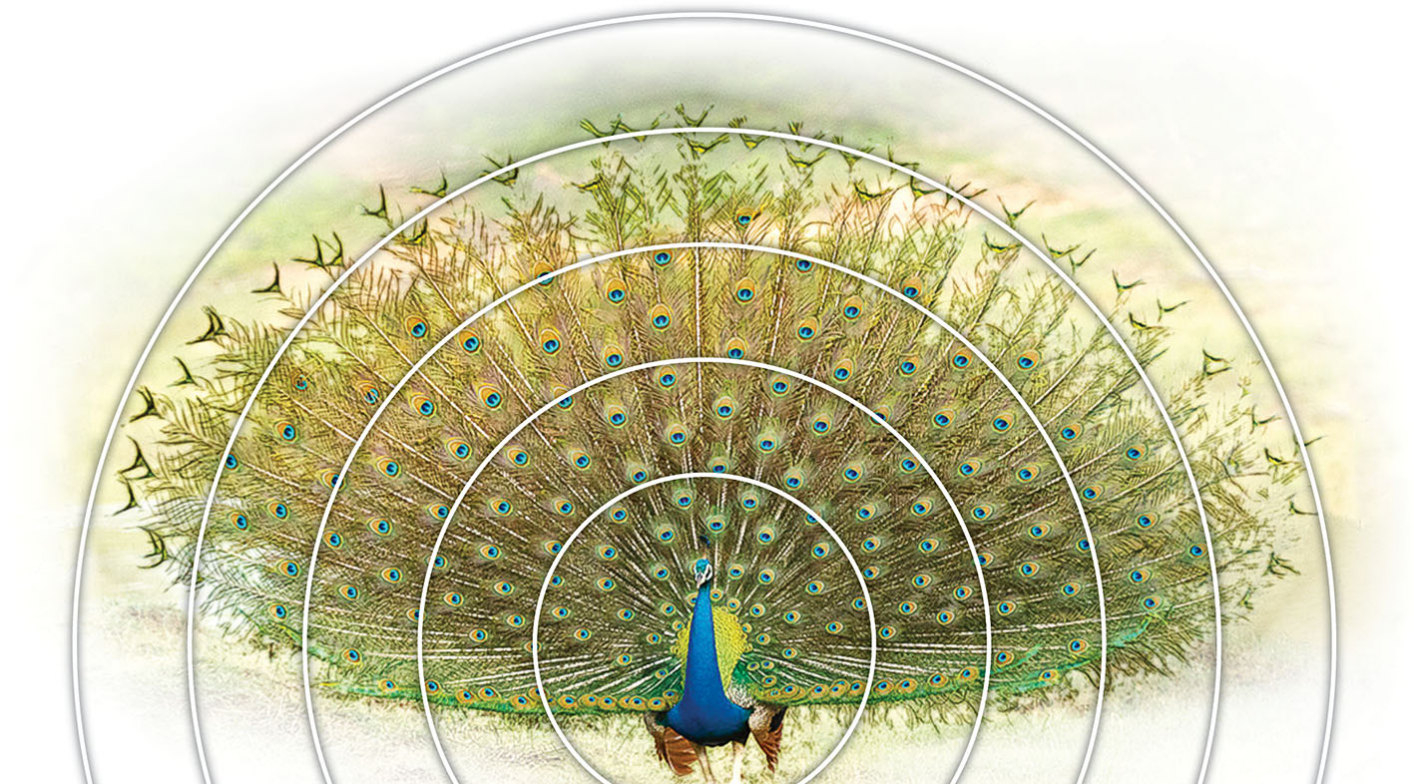
A picture of a peacock tail showing the symmetry of the train and eyespots. Concentric circles show spaced-out eyespots towards the periphery (picture from Wikipedia/ThiminduGoonatil lake from Colombo, Sri Lanka).

To illustrate the eyespot symmetry exhibited by trains in displays ( Figure 5), we made use of Adobe Illustrator’s built-in features (Adobe Photoshop and Adobe InDesign) provided by Graphic Designers’ services at Media Production Services, McMaster University. Briefly, Adobe Illustrator was used to map concentric circles following eyespot distribution on the train in the display by using Figure 1 as a template. These sets of concentric circles were used as the basis for text paths to determine how eyespots may distribute themselves upon the unfolding of the train for display.

To simulate and illustrate the train’s eyespot distribution, we used the following observations and/or assumptions: (1) Based on our overall observations (7 *P. cristatus*, and 1 *P. muticus*), we concluded that each cell on the uropygium ( Figure 1B) represents the base of a corresponding feather; (2) we used a row of 10 or 11 dots in the shape of the (oval) uropygium to represent train feathers but we used a flat and not a convex surface as the latter was not possible. In the result section we discuss why using a convex surface in the simulation would not have changed the outcome; (3) In line with our observation ( Figure 1), we used an arbitrary constant distance between spots within a row and we progressively increased the size of the dot spots between rows, from the bottom (newest) to the top (oldest) feathers, to represent the size of the eyespots as seen on the train; (4) We assumed that each dot represents an eyespot at the end of the feather which is supported by our observation; and (5) we only simulated 2-dimensional position of eyespot distribution and not their three dimensional distribution on the train surface. We simulated four spatial feather arrangements: 10/11 zigzag, 10/11 parallel, 10/10 zigzag, and 11/11 zigzag ( Figure 5). The feather grid was expanded bilaterally and symmetrically to mimic the animal’s train expansion. It is important to point out that the simulated surface shown in Figure 5 represents the front, flat face of the train.

## Results

### The origin of bilateral symmetry in the peacock’s train

**Figure 2.**
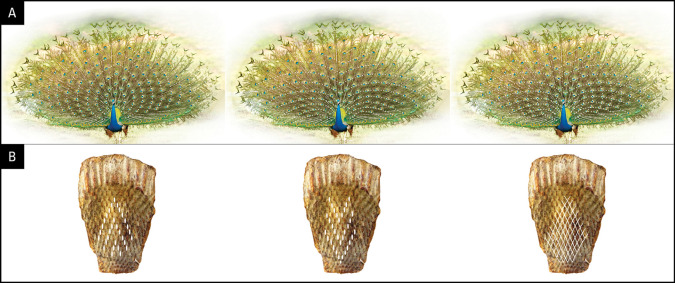
Geometrical designs and the symmetry of the train (A) and the position of follicles on the anchor plate (B). (Peacock picture from Wikipedia/ThiminduGoonatil lake from Colombo, Sri Lanka).

Before presenting our results, it would be important to lay out our expectations that we had before we had seen any museum specimens. Based on our preliminary observation of the structural complexity and the bilateral symmetry of the peacock train ( Figures 1, 2) we had expected to find structural patterns of development which would not allow for intrinsic variation in train morphology. Our results support our expectations.

**Figure 3.**
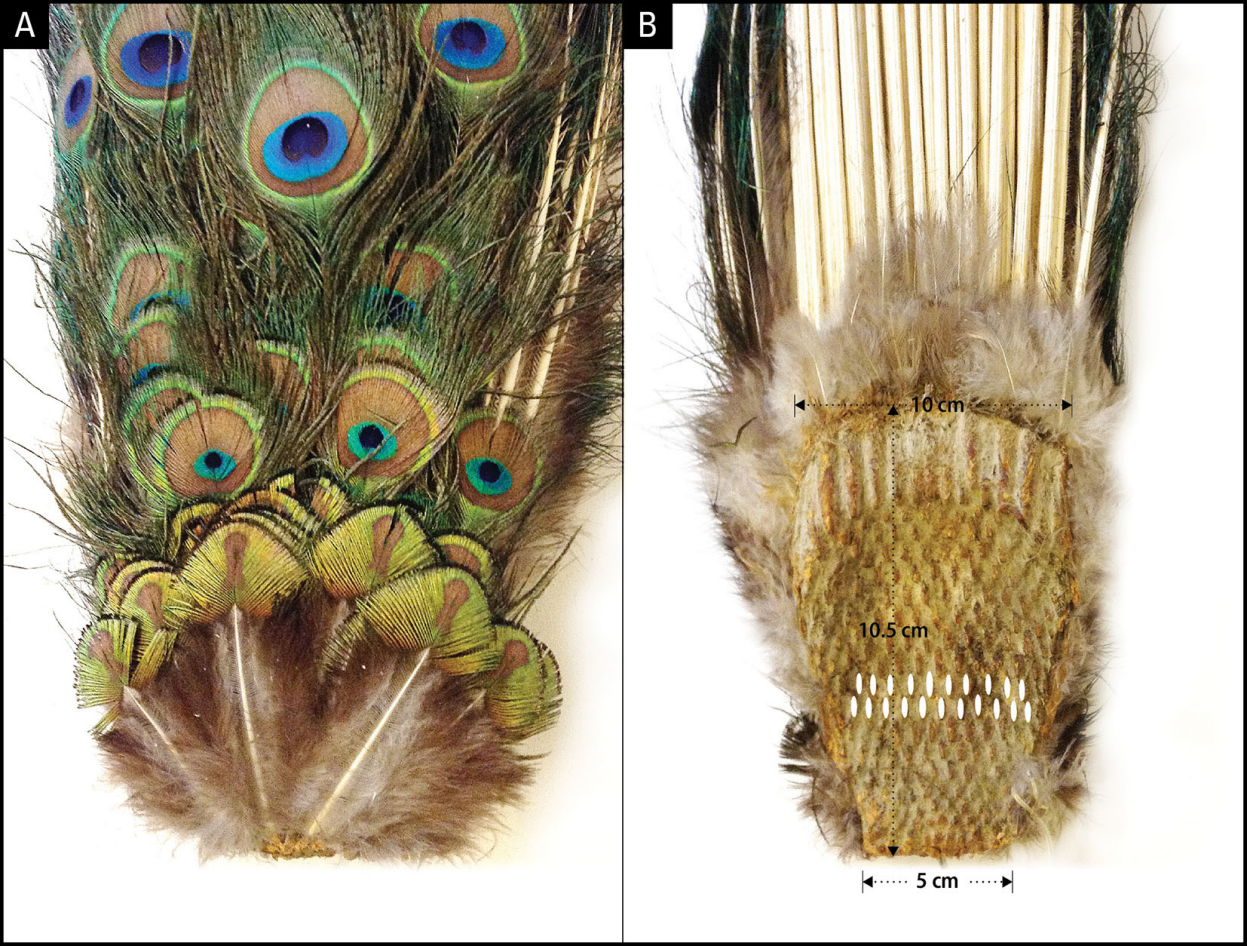
Dorsal and ventral side of uropygium. A: Dorsal side showing the newest rows of feathers at the bottom; B: Ventral side depicting alternating rows of 10 or 11 feather cell imprints. The smallest feather row at the bottom of panel A corresponds to the smallest row of follicles at the bottom of panel B. The top 3-5 rows depending on the age of the animal give rise to fishtail feathers and the rest eyespot feathers.

To uncover the anatomical development plan that determines the bilateral symmetry of eyespots on the train, we examined the uropygium (N = 6) of museum specimens. The uropygium is a fleshy and bony structure at the posterior extremity of a bird’s body that supports the tail feathers. Tail feathers are attached to the dorsal (convex) side of the oval-shaped uropygium, which serves as an anchor plate ( Figure 3). The ventral (concave) side of the anchor plate exhibits several interesting features: 1) It has basal impressions of tightly-packed, parallel rows of feather follicle insertion points; 2) The feather follicles grow progressively larger from the anterior (bottom, younger) to the posterior (top, older) end and are laid out in sequential but alternating rows of 10/11; 3) The posterior feather follicles radiate in an arch, mimicking the fan formation shape of the train in the display. The convexity of the anchor plate makes the train a three-dimensional structure (i.e., an oval trapezoid-shaped dish), such that the feathers are projected outwards at different angles and lengths. Notably, the anchor plate itself appears to be a product of continuous growth and the sequential addition of new rows of feathers each year. The one-to-one correspondence between the pattern of the feather follicles (size, alternate arrangement, and progression from anterior to posterior) on the ventral side of the anchor plate and the symmetrical positions and size of the feathers on the dorsal side is unmistakable and is supported by our observations ( Figure 2).

### Variation in eyespot number

**Figure 4.**
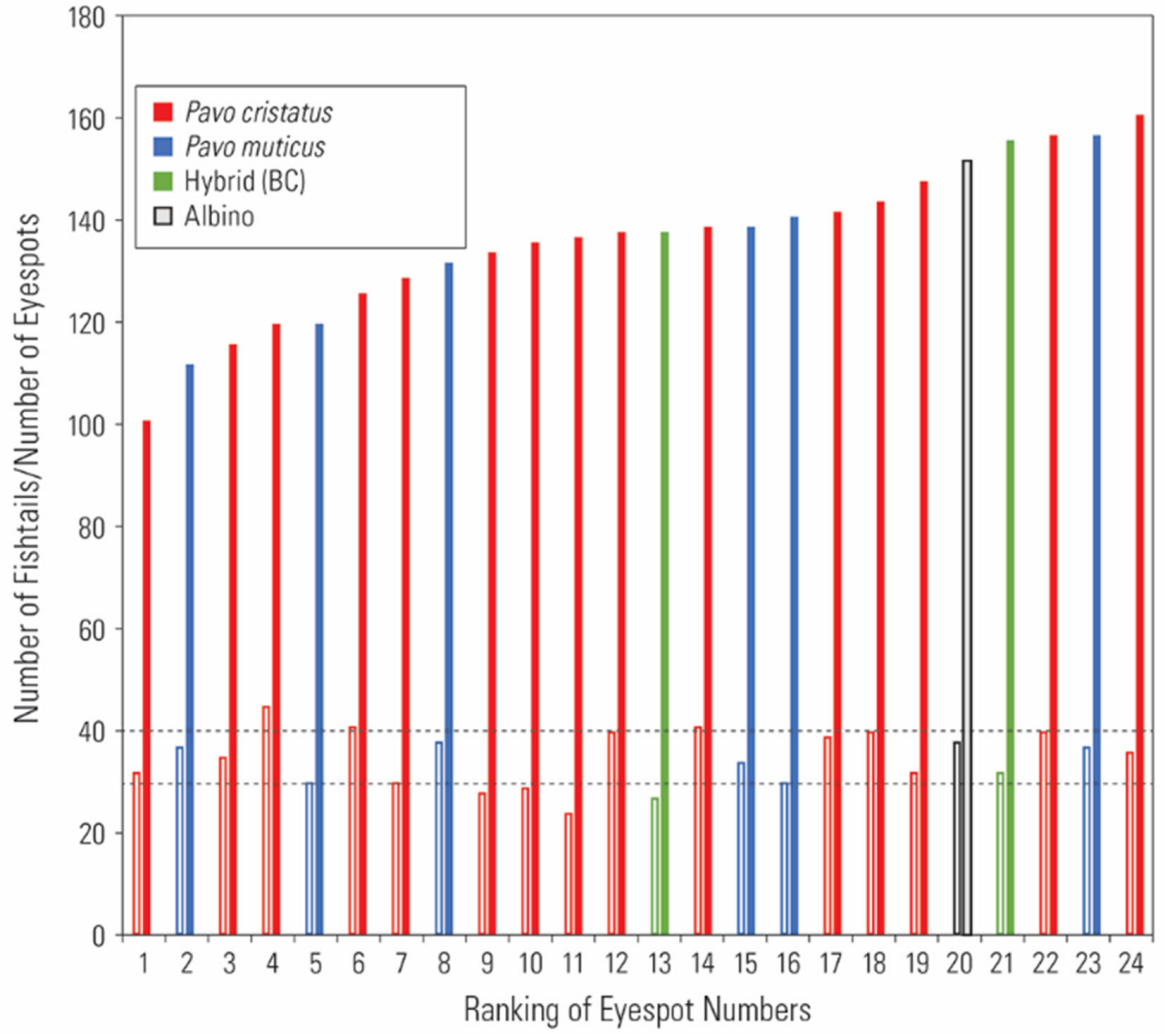
A graph showing independence of eyespot and fishtail feather numbers. Dotted lines represent the number of fishtails (30, 40) consisting of 3 and 4 rows of feathers (details provided in the text). Closed bar: eyespot feathers; open bar: fishtail feathers. The hybrid (BC) is between Bangkok and Cameroon.

The results of morphological variation in the train are summarized in Table 1 and Figure 4. First, the number of feather rows, as determined by the number of follicle rows on the uropygium, varied from 17 to 19 (N = 7), whilst the anterior two to three rows had minor eyespots. The anterior-most rows of eyespots appear minor due to their slow growth and maturity. If one or a few rows of feathers are added each year, then based on the peacock’s longevity of say about 20 years in nature the number of eyespots can reach over 200; however, the late-aged, developmentally immature eyespots (small size and lack of full coloration) will remain insignificant in their effect on female choice.

**Table 1.** Variation in peacock train morphology in *P. cristatus* and *P. muticus.*

	# Feather rows*	# Feathers per row*	# Eyespot feathers	# Fishtail feathers	ESL (cm)	FTL (cm)
*P. cristatus*						
*N*	7	7	8	22	21	21
Mean	18	10/11	129.44	34.42	112.68	134.15
SD	0.63	0	35.70	5.77	25.58	32.17
Range	17–19	10/11	101–161	24–45	47–143	58–160
*P. muticus*						
*N*	1	1	7	7	7	7
Mean	19	10/11	137.50	36.00	101.66	120.16
SD	-	-	14.94	3.52	25.53	29–49
Range	-	-	112–157	30–40	71–128	85–150

N = Number of individuals; ESL: Eyespot feather length, FTL: Fishtail feather length, SD = Standard deviation (for raw data, see Singh, 2021b).

Second, the zigzag arrangement of feathers appeared as an invariant trait in our sample ( Table 1). While this one-to-one correspondence between clearly visible follicle insertion patterns and train morphology may be expected, our data suggest that the developmental plan does not permit random (one feather at a time) variation in eyespot number ( Table 1).

Third, the number of fishtail feathers varied around the modal number of 30 and 40 (N = 21), which is what we would expect if fishtail feathers were produced by three or four rows of feather follicles during development, respectively ( Figure 4). In our museum samples, the highest number of fishtail feathers observed was around 40. However, Figure 1 indicates that the number of fishtail feathers can exceed 50.

Fourth, the number of eyespot feathers varied from 101 to 161 (N = 21), not counting the minor feathers. This variation is attributable to individual variation in terms of age, time of year at capture, and any damage from prior handling at the museum.

Fifth, we speculate that the number of eyespot feathers and feather length are independent traits that vary asymptotically as a function of age.

### Interspecies variation: *P. cristatus* vs. *P. muticus*

We also had access to a small number of *P. muticus* (green peacock) specimens ( *N* = 7) from Southeast Asia ( Table 1; Figure 4). Comparative analyses could provide clues as to whether peacock train morphology is a common feature found across taxa or whether sexual selection and other environmental factors have resulted in different trajectories. In the green peacock, the number of fishtails ranged from 38 to 39 (around the expected mode of 42), while the number of eyespots ranged from 115 to 154.

Data from both species appeared to be homogenous ( Figure 4). Unfortunately, only one green peacock specimen was in pristine condition to enable us to assess the anatomical development pattern of feather follicle rows in the uropygium. As per the blue peacock, feather follicle rows in the green fowl followed the same alternating, 10/11 arrangement, suggesting that this developmental arrangement may be common to other species. Interestingly, similar alternating patterns of train feather arrangements are also found in the wild turkey, *Meleagris ocellata*, a member of the same family (Phasianidae). This observation entertains further research on whether the alternating pattern of feather arrangement might be common across other members of the family.

### Speciation *in-silico *: The origin of the train’s symmetry and complexity

**Figure 5.**
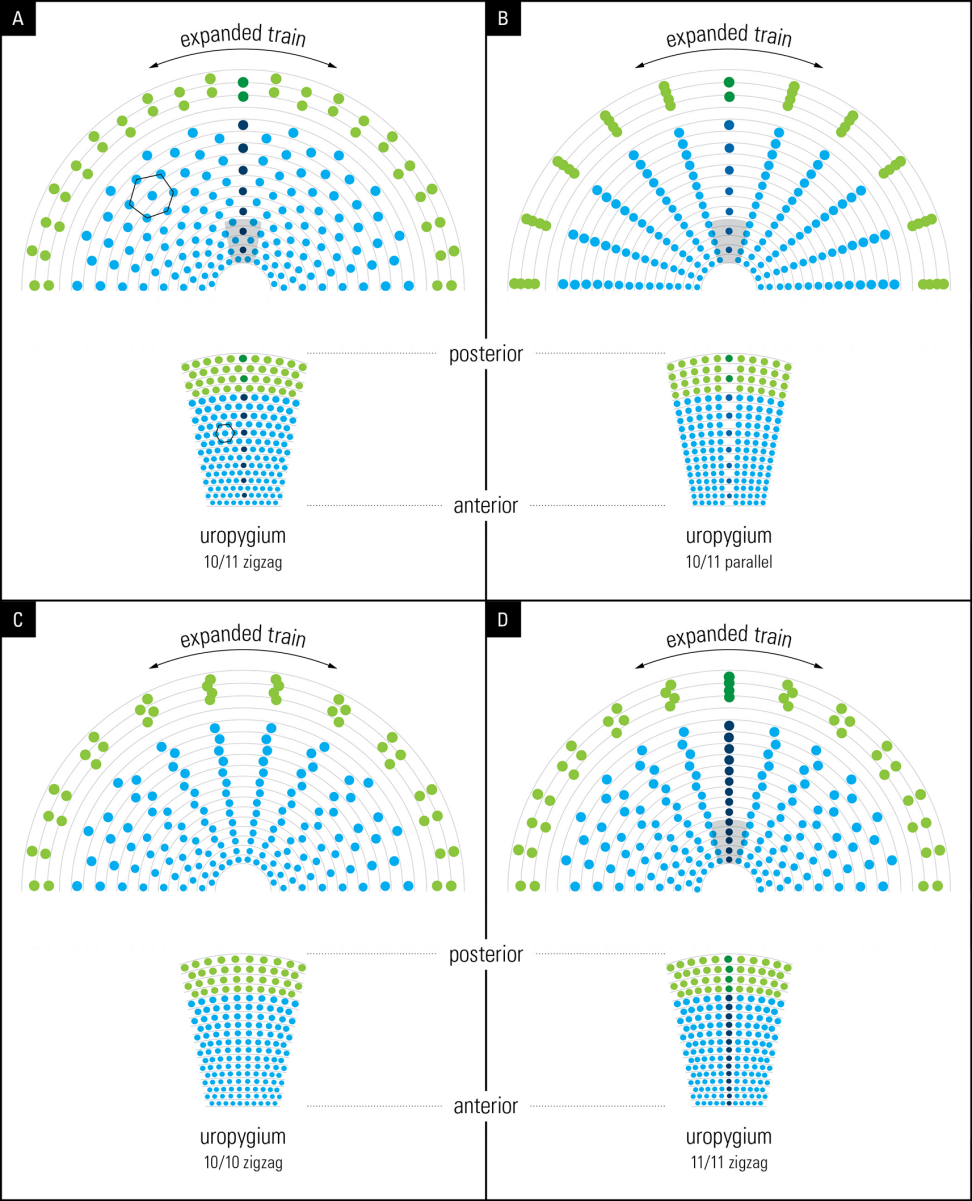
Illustrations from graphic simulations of eyespot symmetry. Layout of feathers on the anchor plate (below) and the resulting eyespot patterns produced by a bilateral symmetrical train (above). The outer spots (green) represent fishtail feathers. (A) 10/11 zigzag pattern; (B) 10/11 parallel pattern; (C) 10/10 zigzag pattern, (D) 11/11 zigzag pattern. Compare the hexagon cellular packing on the uropygium and the expanded train in A with that seen on the animal’s train in Figure 1 (for details see the text).

We investigated the significance of the arrangement of feathers in rows of 10/11 and their “zigzag” alignment ( Figure 2) by simulation (for details, see Methods). To achieve this, briefly, we used graphic design software to reconstruct the unfolding of the peacock’s open train based on the developmental layout of the feather follicle insertion points observed in the anchor plate ( Figure 5, left). Remarkably, our reconstruction yielded an eyespot distribution that is very similar to that observed on the peacock train (compare Figure 5A to Figure 2), with five feathers on either side of the mid-feather in each row. As expected, we found a one-to-one correspondence between the condensed, developmental–anatomical layout of the feather follicle insertion points on the uropygium and the symmetrical distribution of eyespots on the train. It is important to point out that the zigzag arrangement of cells, when spread out uniformly, gives rise to the well-known hexagon cellular packing (compare Figure 5A and Figure 1). We did not simulate a convex surface as this was not possible, however doing so would not have changed the distribution pattern (hexagonal) of eyespot as we were simulating 2-D image of eyespots distribution as seen from front and not their 3-D distribution patterns. In other words, the simulation results presented in Figure 5 show what the imprint/picture would look like if an expanded train were compressed on a flat surface. This is what we would see from a distance.

We further explored what the eyespots distribution pattern would be like if the feathers were arranged in 10/11 parallel rows instead of the zigzag arrangement that we observed in this study. Notably, the train eyespot pattern that we obtained is remarkably different from the 10/11 zigzag pattern. Our reconstruction of a parallel arrangement yielded a palm-leaf-like pattern that fanned out in parallel rows of eyespots rather than the pattern observed in a peacock’s train on display ( Figure 5B). While both patterns are striking, the 10/11 zigzag arrangement yields a denser and uniformly symmetrical arrangement of eyespots, as seen in the animal.

Because the above results raised the question of why 10 or 11 rows were observed, we simulated a 10/10 parallel and 11/11 parallel pattern; the results are shown in Figures 5C and D. Although the 10/10 and 11/11 patterns were like the 10/11 parallel pattern, they showed certain interesting differences. While the feathers lined up in equally spaced, straight lines over the entire span in the 10/11 parallel pattern, the 10/10 and 11/11 parallel patterns resulted in the equal spacing of individual eyespots towards the end of the train, with wavy lines in the center. It is obvious that it was not the zigzag pattern itself but the zigzag pattern of 10/11 that gave rise to the uniform distribution of eyespots on the train. The 10/11 parallel pattern may not be developmentally possible.

### A rudimentary model of eyespot development

We were motivated by the superficial but interesting structural similarity between the iridescence patterning of the eyespots and that of the body plan of the whole animal with a fully expanded train. The entire peacock resembles one giant eyespot in the sense that it boasts a deep-blue body surrounded by a dense zone of bluish-green eyespots, which superficially corresponds to an individual eyespot with its deep-blue inner circle surrounded by radiating oval rings of mixed colors. The same pattern of blue can be observed in the peacock’s crest crown also (data not shown). The blue-green color patch has been shown to be the most important color variables affecting female choice ( Loyau et al., 2007; Dakin and Montgomerie, 2013). Interestingly, Dakin and Montgomerie (2013) point out that “the influence of the other eyespot colors on male success is minimal raising questions about their function.” The similarity between the eyespot color patterns and the peacock’s overall body color suggests the role that constraints play on how traits are made by development and shaped by selection ( Malagón et al., 2014; Ho et al., 2018).

**Figure 6.**
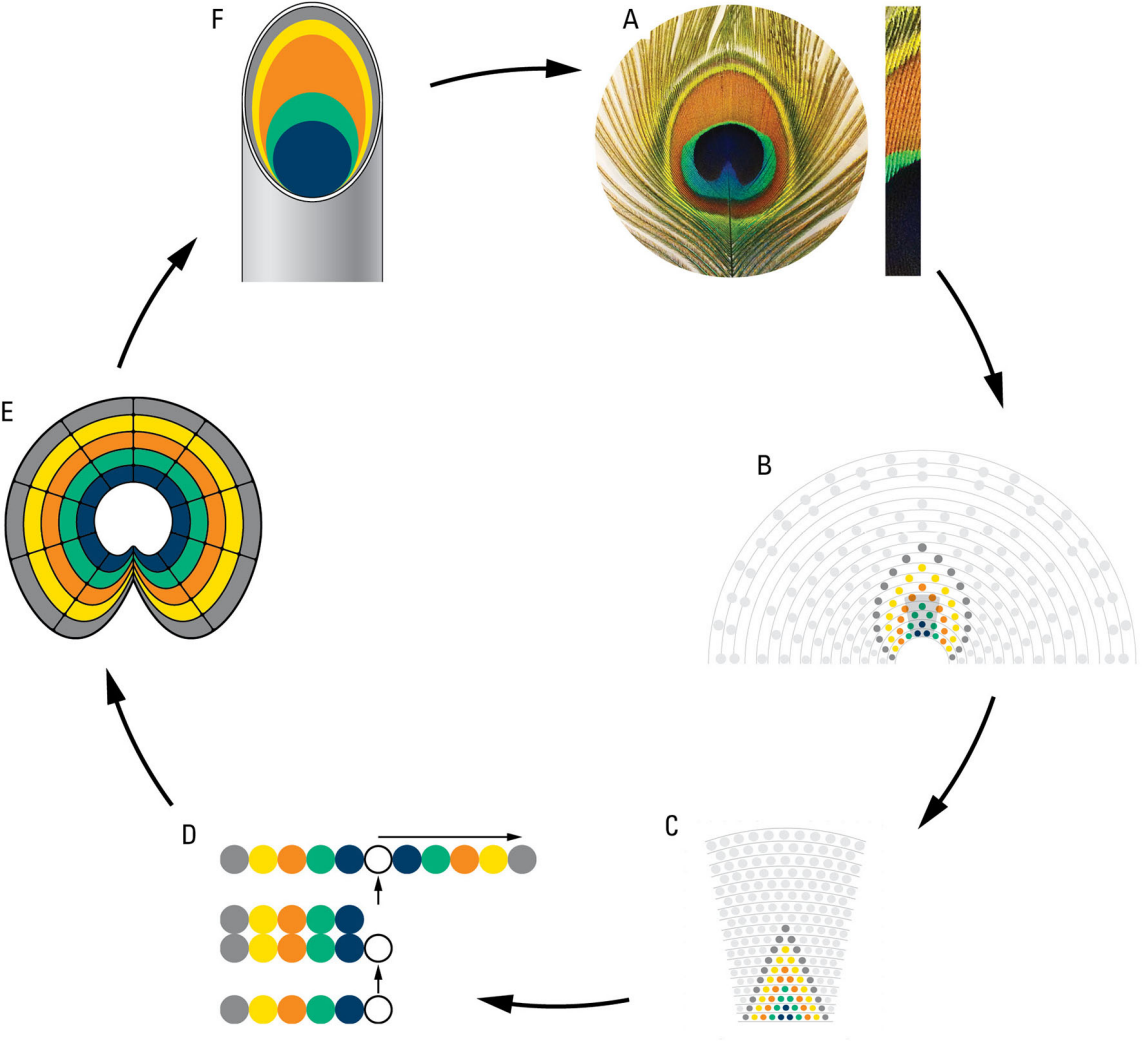
A diagrammatic representation of a theoretical plan of eyespot development as deduced from eyespot to follicles to eyespot. A. an eyespot and a bunch of eyespot fine feathers showing their multi-colored structures; B. modelling of eyespot rings using rings observed on the train; C. projection of train rings on the uropygium; D. cellular processes showing origination of 5 structurally different feather follicles, and their inverted tandem duplication; E. cell growth and invagination of a concentric ring of “stem cells” giving rise to eyespot; F. a peacock feather showing color component of eyespot fine feathers (for details see the text).

We hypothesized that the rings of eyespots may be determined by the same cellular plans as the eyespot arrangements on the train. We took advantage of the similarity between the rings on the individual eyespots and the spatial eyespot rings traced on the train and using reversed logic modeled/walked backward from the eyespot to feather follicles and back to the eyespot ( Figure 6). Starting from each eyespot ( Figure 6A) we inferred colored rings on the train following the pattern seen in one eyespot ( Figure 6B) and from there on to the follicle rows on the uropygium ( Figure 6C). We inferred that the five feather follicles on each side of the central feather make an inverted palindrome and correspond to the five different structural colors seen in the eyespot ( Figure 6D). From this we inferred follicles growth and epidermal invagination giving rise to a concentric ring of the “stem cells” ( Figure 6E) inside the feathers which expand and create the eyespot ring pattern ( Figure 6F). The results show that same cellular mechanisms determine both the eyespot arrangements of the train and the color rings of the eyespots.

## Discussion

The peacock’s train is a complex structure, with the upper train coverts comprising a diverse variety of feather types, each varying in structure, iridescence level, color pattern and symmetry (see Lillie, 1942 and Manning and Hartley, 1991 for details). Mating success of peacocks has been shown to be affected by eyespots and train size but it has not been possible to determine if one or both affect mating success directly ( Petrie et al., 1991; Petrie and Halliday, 1994; Yasmin and Yahya, 1996; Loyau et al., 2005; Harikrishnan et al., 2010). Physically reducing the number of eyespots has a negative effect on mating success ( Petrie and Halliday, 1994); however, Dakin and Montgomerie (2011) showed that females were insensitive to removal of as many as 20% of the eyespots before showing any effect. In the following, after showing that eyespot number and train length are intricately related, and that male vigor is an important factor affecting mating, we present a multimodal model of female choice based on a complex trait combining both train length *and* eyespots ( Andersson, 1994; Candolin, 2003; Bro-Jørgensen, 2010; Mitoyen et al., 2019).

### The zigzag feather pattern explains the train’s symmetry and complexity

The zigzag pattern of feather formation observed from the peacock’s uropygium was able to explain both the eyespot symmetry of the train and the color rings of the eyespots. The latter was shown by using reversed logic and walking backward from the eyespot ring patterns to eyespot symmetry on the train and back to the eyespot ( Figure 6). This exercise was important because it shows that the origin of eyespots is embedded in the overall developmental body plans. This is supported by the fact that Albino peacocks have all the structural details of eyespots except that they lack iridescence. If the origins of eyespots were not in direct response to female choice, it would explain why peahens respond only to the eyespots’ blue-green color which also happen to be the predominant color of the peacock body ( Loyau et al., 2007; Dakin and Montgomerie, 2013). As to the function of the rest of the eyespot colors ( Dakin and Montgomerie, 2013), they may have no function in mate choice and they are there because of developmental constraint; the five primordial eyespot cells giving rise to the five colors go together.

The primordial feather follicles arising on the uropygium in rows of 10/11 are arranged in a zigzag manner. It is this zigzag arrangement of 10/11 that creates the complex hexagonal arrangement of eyespots in the peacock’s tail, and thus its complexity and beauty ( Figure 5, upper left). Hexagonal arrangement is a well-known feature of cellular arrangement in developmental biology ( Sengel, 1976; Classen et al., 2005). The same rule of zigzag arrangement between feather follicles that leads to the complex distribution of eyespots on the train also applies to cellular arrangement of cell lineages within feathers, giving rise to the rings of the eyespot. The qualitative annual growth of eyespot number in rows of 10/11 and the quantitative growth of train length and, essentially, of the former’s dependence on the latter for its presentation and display to females would make the two traits inseparable in their effects on female choice.

### Pattern of feather development constrains eyespot variation

The concept of mutation and variation applies to individual genes or traits leading to variation between individuals. Peacock feathers or eyespots are different kinds of traits in that they do not arise one feather or one eyespot at a time; they are developmental traits and arise in set(s) 10 or 11 annually and this creates constraints on the amount and pattern of eyespot variation observed in populations. As a result, all variation in the total number feathers or eyespots resides between age cohorts and none within age cohorts.

There are two aspects of development that constrain eyespot variation: bilateral symmetry and asymptotic tail growth. The anatomical arrangement and bilateral symmetry of eyespot feathers preclude random genetic variation by addition of single feather at a time and the only apparent source of variation is the annual addition of new feathers in the breeding seasons. Thus, as Takahashi et al. (2008) and Dakin and Montgomerie (2011) found, most males produce approximately 165–170 eyespots, and the small amount of eyespot number variation that exists within and between populations is due to extrinsic factors such as breakage or damage from predation. The actual number of eyespots can exceed 200 ( Figure 3) but many of them would be too small to be visible through experimental photography or to be effective to illicit female response. The idea that the maximum eyespot number in adult animals is invariant within and across populations is consistent with the annual addition of new feathers (10 or 11) to the total that increases asymptotically ( Manning, 1989). It is important to point out that the lack of variation in eyespot number applies to the lifetime total number for individuals within age class and not to the population, which may contain eyespot variation arising from different age classes.

Asymptotic tail growth is the result of aging, loss of vigor or physical strength and diminishing return in fitness accruing from tail growth. After a male has reached his peak reproductive age and achieved the threshold train size to begin mating, his mating success will increase as a function of both the size of his train and his physical strength in mounting a display. But with declining vigor with age and competition from younger males, the individual fitness function will plateau and decline despite large train size. At this point, the law of diminishing returns will commence, and each additional row of feathers will grow slowly, making them look minor, and exert a minimal effect on the feather length and fitness function. Aging males will lack physical strength reducing their changes of securing mates. This is supported by the evidence that females “do not always prefer to mate with older males” ( Petrie, 1993). Moreover, limitations with respect to maximum eyespot number and the lack of correlation between train length and mating success may—as suggested by Takahashi et al. (2008)—indicate that these traits have reached a threshold. And since males do vary in mating success ( Petrie et al., 1991; Yasmin and Yahya, 1996; Loyau et al., 2008), this variation cannot be due to variation in eyespot numbers and other factors may be involved.

### Implication for sexual selection theories and a hypothesis

*Peacock females choose mates on the basis of train size, vigor, and beauty*

Sexual selection studies fall into two groups- a Darwinian framework as suggested by Fisher (1930) and a non-Darwinian framework, the Handicap Principle, suggested by Zahavi (1975). Fisher argued that if females evolve a preference for a conspicuous male trait, for any reason, their offsprings will inherit their preference as well as their fathers’ ornaments. This process, known as runaway selection, will escalate through positive feedback (self-reinforcement) so that sexual ornament will become larger and larger until their negative effects on survival exceed the benefit of attracting mates, creating a trade-off between natural selection and sexual selection. Under the handicap principle, highly exaggerated traits are a handicap, costly (wasteful) signals to covey reliable or “honest” indication of “male quality” ( Zahavi, 1975; Grafen, 1990a, b; Gadagkar, 2003). The “wasteful” connotations of the handicap principle has raised criticism and many studies of mate choice evolution have been done to explore for Darwinian alternatives to the handicap principle (e.g. see Penn and Számadó, 2020; Fromhage and Henshaw, 2022; Henshaw et al., 2022).

Mate search is a highly variable trait and neither of the above two alternatives would appear to apply to the peacock’s tail. Peacock’s tail is clearly not a handicap as there is no way for a cheater to succeed without developing eyespots and a train. All males develop eyespots and a train without exception and all through life. The trains are critical to males’ success in obtaining mates and thus critical to their reproductive fitness.

Peacock’s tail is also not a typical Fisherian ornamental trait. Based on what we know there is no evidence that peahens are paying much attention to the eyespots but the trains are used in male-male competition. The maladaptation of the long tail is clearly the consequence of the adaptation of the train. The maladaptation of the peacock’s long tail may be exaggerated. During the breeding season males are likely to be protected by the lek, and during the non-breeding seasons males shed their feathers. Also, the animals with long tails and prone to predation are likely to be old and past their reproductive peak and are less likely to suffer from fitness loss.

The peacock’s long tail appears to be an unusual case of simultaneous “intra-sex” and “inter-sex” sexual selection: peacocks use their trains to establish male dominance ( Huxley, 1938; Hingston, 1933) much like the deer with their antlers except that, unlike the deer, peahens do the choosing. In this respect, variation in vigor may be an important factor as a younger youthful male with fewer eyespots may outperform an older and bigger male with more eyespots ( Petrie, 1993).

For peacock, we can consider a two-stage model where females are attracted by male size (train size) from a distance, and exercise mate choice based on eyespot beauty, vigor, and behavior from close proximity. Such a model would be able to explain some of the contradictory results between different studies that are based on single variables. It may explain, for example, why females were insensitive to removal of as much as 20% of the eyespots before showing any effect in their behavior ( Petrie and Halliday, 1994; Dakin and Montgomerie, 2011).

*Why females evolve preferences for showy traits - a resolution of the lek paradox*

Neither runaway selection theory nor handicap principle provide an answer to why females initially evolve preference for showy traits. A plausible answer is provided by considering two aspects of the female choice: *mate location* and *sexual stimulation*. The sexes may not always live close to each other and find mates when they need them. This is especially true of sparsely populated species and many showy birds such as the birds of paradise who inhabit dense rainforests. In such species appearance of any showy trait in males would make them more visible to females searching for mates. Locating mates and attracting females’ attention may have been the main reason why females may have started preferring showy traits ( Kirkpatrick and Ryan, 1991). Since showy traits and vigor often go together, the same showy traits that make males visible to females would also evolve to stimulate females’ sexual arousal and choosiness.

It has been argued that since showy traits would evolve rapidly, the genetic variation would get used up in which case why should females choose? This is known as the lek paradox ( Pomiankowski and Moller, 1995; Petrie, 2021). The loss of heritable genetic variation as a result of directional selection from choosiness may not be a problem as traits under strong and persistent selection pressure as well as condition-dependent traits ( Møller and Petrie, 2002) may tap in the hidden variation locked up in developmental pathways ( Singh, 2021b). But even if all genetic and developmental variation, segregating or hidden, was lost due to directional selection, male display of showy traits and female sexual arousal would get locked up and become a part of the mating rituals and males would keep displaying and females keep choosing mates. Mate choice affecting showy traits would be maintained by purifying selection and they would keep serving females as a source of sexual attraction and stimulation. All discussions of sexual selection have focused on why females choose in relation to fitness- directly or indirectly. This may not be true. Females may be choosing mates on the basis of traits affecting sexual attraction and stimulation, not fitness. This provides a parsimonious resolution of the lek paradox ( Pomiankowski and Moller, 1995).

*Non-sexual selection theories of peacock’s bright coloration*

All the above scenarios are in relation to the peacock tail being the product of sexual selection. Alternate explanations have been presented whereby bright coloration in animals is treated as an expression of protection against fear ( Hingston, 1933). Peacock’s impressive train has been explained by the “aposematic hypothesis” ( Jordania, 2021) which proposes that rather than concealing themselves some species use conspicuous traits including size, sound, color, fluffiness, odor, and threatening gestures to ward off predators. Another study has taken on the theme of ‘food and sex go together’ and has proposed the “food courting hypothesis” ( Jacobs, 1999) suggesting that ocelli remind peahens blueberries!

### Limitation and future work

The small sample size of museum specimens used in this study may raise concerns about the validity of our results. The most important results of this study, shown in Figure 5 and Figure 6, are qualitative and/or based on theoretical modelling, and are unaffected by sample size. Simulation is a form of hypothesis testing, but it lacks direct anatomical testing which is outside the scope of our lab. The zigzag arrangement of feathers and its effect on the train’s symmetry and complexity ( Figure 5) were simulated and shown to conform to observations from this work and at least the number and the symmetry of feathers is supported by previous work ( Dakin and Montgomerie, 2011). The rudimentary model of eyespot development ( Figure 6) would also need to be investigated by developmental work. Future tests can involve (1) anatomical dissection of live animals to test the number and the arrangement of feathers as well as the correspondence between feathers and feather buds, (2) developmental work on young peacocks to test the annual addition of feather rows as well as feather growth as a function of age, (3) further work on the line of Dakin and Montgomerie (2011) to test the effect of varying eyespot numbers on female choice, and (4) work to test the effect of variation in male vigor as a function of age on female choice.

## Conclusions

To summarize, in this study we showed that (1) a zigzag pattern of feather formation affects the bilateral symmetry, eyespot complexity, and structural beauty of the peacock’s tail; (2) the same zigzag pattern can, remarkably, also explain the colorful rings of the eyespot; and that (3) the zigzag pattern would preclude intrinsic variation in the total number of eyespots among adult individuals. The only source of variation in eyespot number would be the annual addition of eyespot feathers, in rows of 10 or 11, giving rise to variation between the age classes.

These results led us to three insights that would help explain conflicting results between studies in literature. First, eyespot number and train size are developmentally connected such that eyespots do not drive train length: it is the other way around—it is the feather/train growth that pulls eyespots up, spreads them out, and makes them visible to the female from afar. Second, we showed that eyespot number and male tail growth both have asymptotic functions, which means that later stage addition of feathers would lead to minor and ineffective eyespots with no added benefit to males. Finally, we argue that male vigor is a crucial factor modulating the effects of male size and beauty. Two males can have the same number of eyespots but differ in their age and vigor. Taken together, these insights can explain many of the conflicting results reported from different studies in the past.

Based on our results, we propose a two-stage, multimodal model of female choice based on male size, beauty, and vigor and suggest that females may not be using eyespot number or feather train size but a combined complex trait, possibly a “blue-green” train ( Loyau et al., 2007; Dakin and Montgomerie, 2013), as a basis of mate choice. Since blue-green is also the color of the male body it would appear as if peahens are not much affected by the details of the eyespots “beauty”. Females may be choosing the tallest, the most vigorous, and the most “beautiful” males for mates. Our results solve the problem of the relationship between eyespot number and tail length and provide a new perspective on the role of male size, vigor, and beauty in the evolution of female mate choice.

## Author contributions

RSS: Development of concept, collection of data, theoretical–developmental modelling, critical review of sexual selection theories, preparation of the manuscript, and financial support; SJ: literature review, critical analysis of sexual selection theories, data analysis, and preparation of the manuscript.

## Ethics and consent

Ethical approval and written consent were not required.

## Data Availability

Dryad: A long tail of truth and beauty: a simple rule of pattern formation explains symmetry, complexity and beauty in the peacock’s tail, https://doi.org/10.5061/dryad.1g1jwstwg ( Singh, 2021a). Data is available under the terms of the (CC0 1.0) Public Domain Dedication license.
