# Supplementary material for: A long tail of truth and beauty: A zigzag pattern of feather formation determines the symmetry, complexity, and beauty of the peacock’s tail
Source: F1000Res. 2025 May 6;13:520. Originally published 2024 May 22. [Version 3] doi: 10.12688/f1000research.149948.3 (PMC12022539; doi:10.12688/f1000research.149948.3)
Supplement: Peer review history

# Reviewer report: Reviewer response for version 3

**DOI:** 10.5256/f1000research.181505.r383122

**Published:** 2025-09-11

**Authors:** Jutta Schneider

**Affiliations:** 1 University of Hamburg, Hamburg, Germany

I am satisfied with the changes and recommend the article for indexing.

Is the work clearly and accurately presented and does it cite the current literature?

Partly

If applicable, is the statistical analysis and its interpretation appropriate?

Not applicable

Are all the source data underlying the results available to ensure full reproducibility?

Yes

Is the study design appropriate and is the work technically sound?

Yes

Are the conclusions drawn adequately supported by the results?

Partly

Are sufficient details of methods and analysis provided to allow replication by others?

Partly

Reviewer Expertise:

NA

I confirm that I have read this submission and believe that I have an appropriate level of expertise to confirm that it is of an acceptable scientific standard.

# Response

**Published:** 2025-09-11

**Authors:** Rama Singh

**Affiliations:** McMaster University, Canada

I sincerely appreciate and thank Dr. Schneider for her approval of the paper. While requiring two reviewers approvals may not be too high a bar to publish a paper, it can be a too low bar to reject a good idea.

 I am happy with the outcome.

 Rama Singh

# Reviewer report: Reviewer response for version 2

**DOI:** 10.5256/f1000research.178029.r372562

**Published:** 2025-04-24

**Authors:** Jutta Schneider

**Affiliations:** 1 University of Hamburg, Hamburg, Germany

This interesting paper provides relevant information about the morphological principles underlying the development of the features of the peacock train. The results suggest that traits relevant for female choice, namely eyespot number and symmetry are developmentally constrained. This finding allows for some novel perspectives on the function of the peacock train although I doubt that “it can solve longstanding puzzles concerning female choice in peacocks”. The manuscript was revised based on the comments of two reviewers. However, I found the paper in parts still hard to follow and suggest some more work on clarifying structure and logic. I would further recommend cautiousness in interpretating the results.

 Details:

 The authors used a species mix of museum specimen (n=21) and quantified several features of the tail, including the arrangement of eyespot feathers and numbers of eyespots, as well as fishtail feathers. The uropygium from which the feathers originate was closely inspected and revealed important insights. The novel finding is that the symmetry of the train can be explained by a zigzag patterning of feather imprints on the uropygium. This was shown using clever simulations of feather follicles patterns. This part including figures 2 and figure 5 is interesting and well explained. The authors suggest that the patterning of the follicles does not permit variation in eye-spot number based on male traits other than age. While I do not doubt the conclusion, I would like to see this logic explained a bit more clearly.

 Table 1 and Figure 4 would benefit from clarifications. To avoid confusion the x-axis of fig 4 should be labelled as ”ranking of eyespot numbers” or just “ranks”. The legends state that fishtails vary around 30+, 40+ and 50+ but I cannot see a bar that is higher than 45. In the text the author refer to Figure 1 to back the claim that eyespots can exceed 50. However, Figure 1 just shows a single picture copied from Wikipedia. How much can be generalised from that? Staying with the paragraph on variation in eyespot number, I wonder how informative a comparison between two species and a hybrid can be, given the small sample sizes? A conclusion based on one green peacock that had an intact uropygium with the 10/11 arrangement seems rather bold. I suggest to at least tone down the statement that this is a “invariant feature across peafowl species”. The entire paragraph would benefit from a revision.

 The paragraph (a rudimentary model) is concerned with the eyespot development is difficult to follow. Maybe I am lacking imagination, but comparing figure 1 to an individual eyespot, I am not sure that I see a close resemblance to the design of individual eyespots.  Given there is a resemblance, the method of using the reversed logic and map the ring patterns on an imaginative uropygium is interesting and does indeed reveal a suggestive similarity. However, a better explanation would be helpful as well as an better justification of why this exercise is important.

 Discussion

 The authors start the discussion explaining the relevance of the train features for female choice and that it is unknown whether mating success depends on number of eyespots or train size or both. The authors claim that their study can fill this knowledge gap. The morphological data and the simulations suggest that train size and eyespot number are not independent traits. While this is an important and convincing interpretation of the data, I would argue that the study cannot draw conclusions about e.g. the role of male vigor for female choice. Furthermore, while I agree that the inspection of the uropygium provides an explanation for the development of the eyespot symmetry, I am not sure about the explanations of the within eyespot ring patterns. The claims derived from the patterns of “cellular arrangement of cell lineages within eyespots” must be explained better for non-specialists like me to understand.

 A paragraph follows on ”implications for sexual selection theories ..”  and I again strongly suggest that the authors stick to what their data permit rather than making broad inferences about behaviour and mate choice. Claims about the handicap principle like this one: “Under this model peacock tail is not only not maladaptive, but it also enables peacocks to survive better” are rather strange. The results do also not permit statements like that: “The results of the present study suggest that neither the runway selection nor the handicap model apply to the case of peacock tail”. The claim “our study brings out several important results that have implications for how the peacock’s attractive train develops and how females choose mates” is only partly true, as no data on female choice are presented here.

 In defence of the handicap principle, I would argue that male age in combination with a large and intact train can be a valuable quality signal if there is variation in how well males age. The authors claim that vigor will decline with age, which in general is reasonable to assume. However, there might be variation in age-dependent vigor, which brings me to the incomplete discussion of the handicap principle. I suggest to rewrite the entire paragraph by using the most recent literature, (see citations).

Is the work clearly and accurately presented and does it cite the current literature?

Partly

If applicable, is the statistical analysis and its interpretation appropriate?

Not applicable

Are all the source data underlying the results available to ensure full reproducibility?

Yes

Is the study design appropriate and is the work technically sound?

Yes

Are the conclusions drawn adequately supported by the results?

Partly

Are sufficient details of methods and analysis provided to allow replication by others?

Partly

Reviewer Expertise:

Behavioural ecology

I confirm that I have read this submission and believe that I have an appropriate level of expertise to confirm that it is of an acceptable scientific standard, however I have significant reservations, as outlined above.

## References

[1] : The balance model of honest sexual signaling. *Evolution* .2022;76(3) : 10.1111/evo.14436 445-454 10.1111/evo.14436 35075645 PMC9303242

[2] : The evolution of mating preferences for genetic attractiveness and quality in the presence of sensory bias. *Proc Natl Acad Sci U S A* .2022;119(33) : 10.1073/pnas.2206262119 e2206262119 10.1073/pnas.2206262119 35939704 PMC9388091

# Response

**Published:** 2025-05-01

**Authors:** Rama Singh

**Affiliations:** McMaster University, Canada

1. Response to Reviewer
  We sincerely thank the reviewer for their critical comments, some of which have made us seriously think - such as the comments made on Fig 6 and the need for justification of why this exercise was done. We have read the comments carefully and have made the following changes in the revised version:
2. Table 1: It  was fixed by adding more information.
3. Figure 4: The dotted line at Y-50 was removed and the X-axis labeling was revised.
4. Constraints on variation: We added a few lines explaining how the variation in feather and eyespot number is developmental and occurs in sets of 10/11  at a time as compared to random mutational variation in other traits.
5. Two- species comparison: The limitation of having only one intact uropygium in *P. muticus* is well taken and the conclusions are toned down.
6. Fig 6: The logic of the exercise behind this figure is explained. We make the point that the origin of eyespots is determined by the same cellular mechanisms as those determining the eyespot distribution on the train. We make the point that eyespots did not originate in direct response to female choice and this may explain why females respond only partially to it- using  the blue-green color which also happen to be the dominant color of the peacock’s body! It does not look like females are making much use of the eyespots’  “beauty”.
7. Role of male vigor in female mate choice: We are surprised that the reviewer questions the role of male vigor by which we simply mean “physical strength”. Males defend their territories and put on display dance. Unless we have misunderstood the reviewer, we think the role of male vigor in mate choice should be obvious.
8. Theories of the mate choice evolution: We agree with the reviewer that description of handicap was incomplete and we have fixed it. We justify the brief discussion of Darwinian (Fisher’s runaway selection) and non-Darwinian (Zahavi’s handicap) as it provides a framework to discuss mechanisms of the evolution of the peacock’s tail. We thank the reviewer for bringing relevant references to our attention.
9. We hope the reviewer likes the revised version and approves it. 
  Thank you. 

# Reviewer report: Reviewer response for version 2

**DOI:** 10.5256/f1000research.178029.r368433

**Published:** 2025-02-28

**Authors:** Joseph Jordania

**Affiliations:** 1 University of Melbourne, Parkville, Australia

I can confirm that I did read the new version of the article and find it much improved! My critical comments do not apply any more.

Is the work clearly and accurately presented and does it cite the current literature?

Partly

If applicable, is the statistical analysis and its interpretation appropriate?

Not applicable

Are all the source data underlying the results available to ensure full reproducibility?

No source data required

Are the conclusions drawn adequately supported by the results?

Yes

Are sufficient details of methods and analysis provided to allow replication by others?

Partly

Reviewer Expertise:

Evolutionary psychology; Behavioural ecology; Animal defence strategies; Evolutionary musicology

I confirm that I have read this submission and believe that I have an appropriate level of expertise to confirm that it is of an acceptable scientific standard.

# Response

**Published:** 2025-02-28

**Authors:** Rama Singh

**Affiliations:** McMaster University, Canada

Thank you, Dr. Jordania, for your approval of the manuscript. Hopefully it will also be approved by Reviewer 2 and get published. 

 Rama Singh

# Reviewer report: Reviewer response for version 1

**DOI:** 10.5256/f1000research.164464.r356816

**Published:** 2025-02-05

**Authors:** Joao Menezes

**Affiliations:** 1 Universidade Federal de São Paulo, São Paulo, Brazil

This manuscript investigates the anatomical basis of the peacock’s train morphology, proposing that feather follicles follow a zigzag pattern that constrains eyespot number and contributes to the train’s symmetry and complexity. Based on observations of museum specimens and graphical reconstructions, the authors argue that train size and eyespot distribution are largely predetermined by developmental constraints, which may explain inconsistencies in past studies of sexual selection in peafowl.

 The authors provides intriguing evidence that train morphology is largely constrained by developmental processes, with important implications for how sexual selection operates in peafowl, a textbook example of mate choice. By demonstrating that eyespot number is relatively fixed in adult males, the authors offer a potential explanation for conflicting findings in past studies on mate selection. Additionally, the use of graphical simulations is an original — and visually appealing — approach for visualizing the structural patterns underlying train symmetry.

 Having said that, while this study seems to present a promising contribution to the sexual selection literature, I found it difficult to fully assess its scientific merit due to the manuscript’s lack of organization and clarity. The way information is presented both within and across sections makes it challenging to follow the logical progression of the study. Below, I outline major issues that, in my view, are preventing an effective communication of the manuscript's findings. I would be happy to assess the further merits of the study in a future, more reader-friendly version.

 *Overall organization*

 The manuscript lacks a clear and logical structure, making it difficult to navigate. This is most noticeable in the way that introduction, methods, results, and discussion are interwoven across the manuscript.

 First, while it is common to provide a brief preview of key findings at the end of the Introduction to help orient the reader, the manuscript dedicates two full paragraphs to presenting and discussing results. Such elaboration should ideally be reserved for the Results and Discussion sections.

 Similarly, the Results section includes substantial content that would be more appropriate in other sections. For example, details on how museum specimens were selected and how graphical simulations were performed should be in the Methods section, yet these details are scattered throughout the Results. Likewise, several sentences contain citations to previous studies, indicating that they belong either in the Introduction or the Discussion. Among many examples, the authors discuss Takahashi et al. (2008) and Dakin and Montgomerie (2011) when interpreting their own findings (something that should be done in the Discussion) and present background information on feather development (which would be better suited to the Introduction).

 I highly recommend reorganizing the manuscript so that background information is confined to the Introduction, Methods are clearly described in a dedicated section (and only there), and Results present only the study’s objective findings (without citations to external literature or interpretations). Such restructuring would make the paper significantly easier to follow and ensure that each section serves its intended purpose.

 *Logical flow and conciseness*

 Within sections, the manuscript is often difficult to follow due to unclear sentence structure, awkward phrasing, and abrupt shifts in focus. Many sentences are unnecessarily complex, often including parenthetical remarks that disrupt readability. For example, the opening sentence of the manuscript includes a long parenthetic note that, while potentially relevant, interrupts the flow of the introduction and could be better placed later in the text. Additionally, logical inconsistencies and imprecise wording make several sentences unclear. For instance, in the Introduction, the phrase starting with “Unlike in other animals…” implies either that peacock’s tail cannot be directly under female choice, or that sexually selected traits in other animals cannot have three aspects — neither of which makes much sense. I chose two examples in the first paragraph because this paragraph is crucial to engaging readers, but these issues are present throughout the manuscript.

 The introduction also frequently presents ideas in a way that lacks explicit connections, making it difficult to follow the logic of the argument. The third paragraph, for example, abruptly moves from discussing previous studies on sexual selection to a description of train symmetry and feather follicle arrangements. The transition between these topics is unclear, and the sudden introduction of figures further disrupts the flow. Particular care should be given to this paragraph because it establishes the goals of the study and therefore is key for full comprehension of the manuscript.

 The manuscript would greatly benefit from more direct and concise writing, with clearer sentence structures, fewer parenthetical remarks, and better transitions between ideas.

Is the work clearly and accurately presented and does it cite the current literature?

Partly

If applicable, is the statistical analysis and its interpretation appropriate?

Not applicable

Are all the source data underlying the results available to ensure full reproducibility?

Yes

Is the study design appropriate and is the work technically sound?

Yes

Are the conclusions drawn adequately supported by the results?

Partly

Are sufficient details of methods and analysis provided to allow replication by others?

Partly

Reviewer Expertise:

animal communication, sexual selection, bioacoustics, ornithology, macroevolution

I confirm that I have read this submission and believe that I have an appropriate level of expertise to confirm that it is of an acceptable scientific standard, however I have significant reservations, as outlined above.

# Response

**Published:** 2025-02-07

**Authors:** Rama Singh

**Affiliations:** McMaster University, Canada

Revisions made in Version 2:

 Reviewer: Dr. Menezes

 We sincerely thank the reviewer for liking the paper and noticing its originality. We see that the reviewer has read the paper very carefully as only a careful reading would pick up all the editorial sloppiness that has crept unnoticed in the manuscript! We have accepted all the suggestions. The paper has gone through thorough editing and cleaning up making it easier to read.

 We again thank the reviewers for their critical comments and hope that the revised paper will get their final Approval.

 Rama Singh

# Reviewer report: Reviewer response for version 1

**DOI:** 10.5256/f1000research.164464.r311984

**Published:** 2024-10-08

**Authors:** Joseph Jordania

**Affiliations:** 1 University of Melbourne, Parkville, Australia

Since the publication of Charles Darwin’s book on human evolution via sexual selection (1871) the striking beauty of the male peafowl (peacock) tail has fascinated generations of scholars. Today there are quite a few hypotheses behind the possible evolutionary reasons of this remarkable phenomenon. But strangely, together with the admiration, this phenomenon suffered one of the longest scholarly neglects from behavioural ecologists. For more than a century after Darwin’s initial hypothesis, scholars did not try to back up Darwin’s hypothesis with any sort of data of experimental or field study. The first study was done in 1991, and we must be grateful to the authors of the pioneer study (Petrie et al. 1991). 

 Unfortunately, the study was very limited in many ways. For example, the researchers studied only one lek (a congregation of males) of 10 males during one month (by the way, the authors of the reviewed article do not mention these limitations). Also, the method employed did not convince everyone – researchers disfigured some of the males’ tail feathers and covered several eyespots to observe the results, and the results appear only after 20 % of the eyespots wee disfigured. 

 It is important to remember the fact (also not mentioned in the reviewed article), that Takahachi et al study was expected to confirm the Petrie et al. 1991 findings with more solid field results. Very unexpectedly for them, the researchers came to a conclusion, that the peahens were totally indifferent to the peacocks’ tail beauty and eyespot numbers, and that generally the tail condition did not correlate with the reproductive success of their bearers. 

 Authors of the reviewed article suggest an original idea that for their all-important “female choice” peahens use several elements of male train in combination. As I know, this idea was expressed for the first time in the published literature. So, my congratulations to the authors.

 At the same time, I would suggest to double-check the crucial premise of the article, (“While a relationship between the feathers’ elaborate features and mating success has been shown…”) this hypothesis is by no means guaranteed. There is a relatively recent publication that suggests that the evolutionary mechanism behind the peacock’s train must be found in natural selection theory, more precisely, on aposematic defense from predators (not sexual selection theory).

 As known to behavioural ecologists, aposematic species do not try to conceal themselves from predators. On the contrary, they try to “announce” their presence by all the possible modalities: they are often very visible by their body size and contrastive colours; they often make constant sounds as they walk and have very loud voice; and they often have a constant body odour and can produce stronger smell if threatened. Finally, when confronted by a potential predator, instead of running away, they try to intimidate the predator by their suddenly increased body size, threatening sounds, gestures, and fearless behaviour. 

 The author of “aposematic hypothesis” (Jordania, 2011, 2021) proposed that apart from huge and brilliant coloured body with many “eyespots” (visual signals), the peacock has a very strong, piercing voice (audio signal), secretes very smelly droppings if handled against its will (olfactory signal), and often does not go away even if a potential danger, for example, a tiger or a leopard, is nearby (behavioural signal, attested by many scholars, starting from George Schaller). So, aposematic hypothesis should be at least critically discussed in the reviewed article.

 In short, in my opinion, the presented article is a welcome addition to the scholarly discussion on this important topic. Therefore, I want say that I support indexing of this article, but with certain modifications and additions, as there is a plenty of room to make the article more informative and methodologically more robust. I will condense my comments to several concrete points:

 (1)     Most importantly, the article misses a mention and discussion of several important publications in the sphere, such as the following works: 

 Ref-1,2,3, 4

 (5)    Merle Jacob on the food courting theory, proposing that ocelli remind females blueberries. See: Jacobs, M. (1999) "A New Look at Darwinian Sexual Selection". NaturalSCIENCE. Heron Publishing.

 (6)     Also, the reviewed article could mention the early works of Huxley and Hingston, who are often mentioned together as they both proposed that peacock train was used to establish dominance among group of males. See: Huxley, J.S. 1938. Darwin's theory of sexual selection and the data subsumed by it in the light of recent research. American Naturalist 72:416-433. Also see: Hingston, R.W.G. 1933. The meaning of animal colour and adornment. Edward Arnold, London.

 I am by no means suggesting the authors of the reviewed article to alter in any way their original view on the subject, but I do believe that they should critically discuss the above-mentioned views on the subject. This will make their article more comprehensive and updated with contemporary literature on the subject.

Is the work clearly and accurately presented and does it cite the current literature?

Partly

If applicable, is the statistical analysis and its interpretation appropriate?

Not applicable

Are all the source data underlying the results available to ensure full reproducibility?

No source data required

Are the conclusions drawn adequately supported by the results?

Yes

Are sufficient details of methods and analysis provided to allow replication by others?

Partly

Reviewer Expertise:

Evolutionary psychology; Behavioural ecology; Animal defence strategies; Evolutionary musicology;

I confirm that I have read this submission and believe that I have an appropriate level of expertise to confirm that it is of an acceptable scientific standard, however I have significant reservations, as outlined above.

## References

[1] : Mate selection-a selection for a handicap. *J Theor Biol* .1975;53(1) : 10.1016/0022-5193(75)90111-3 205-14 10.1016/0022-5193(75)90111-3 1195756

[2] : The Handicap Principle: how an erroneous hypothesis became a scientific principle. *Biol Rev Camb Philos Soc* .2020;95(1) : 10.1111/brv.12563 267-290 10.1111/brv.12563 31642592 PMC7004190

[3] : Eye for an eyespot: how iridescent plumage ocelli influence peacock mating success. *Behavioral Ecology* .2013;24(5) : 10.1093/beheco/art045 1048-1057 10.1093/beheco/art045

[4] : Can there be an Alternative Evolutionary Reason Behind the Peacock’s Impressive Train?. *Academia Letters* .2021; 10.20935/AL3534 10.20935/AL3534

# Response

**Published:** 2024-10-11

**Authors:** Rama Singh

**Affiliations:** McMaster University, Canada

Response to Reviewer (Dr. Joseph Jordania):

 A peacock manuscript coming from a fruit fly lab must surely appear as odd. Our long term research on sex and reproduction related genes and their role in evolution and speciation led our interest to sexual selection and mate choice. This gave rise important discoveries such as: the *faster evolution of sex and reproduction related genes* (Haerty et al. 2007. Genetics177: 1321-1335), *a theory of male drive* (Singh and Kulathinal 2005. BioEssays 27: 518-25; Jagadeeshan et al. 2015. PLoS ONE. doi:10.1371/journal.pone.0144672), and the *mate choice theory of menopause* (PLoS Com. Biol. https://doi.org/10.1371/journal.pcbi.1003092).

 A casual observation of a picture of the peacock’s train in a Press Release from our lab drew our attention to the stunning geometric patterns of eyespots formation on the peacock’s train. This led to over three years of investigation that is described in the article under review. We discovered a simple developmental rule behind the symmetry of the train and we thought our findings had important bearing on the theory of sexual selection and female choice. We wrote the paper, submitted it to the journal, and eagerly waited for the response. We got the response and we were disappointed.

 The main problem was that while the data and the results of the study had to do with *anatomical development*, their implications were in the area of evolution and sexual selection.  When the paper was submitted to developmental journals, we were told to submit it to evolution journals or to anatomy. When we submitted to evolutionary journals, we were told they had difficulty finding reviewers and when they did find reviewers, they had very little to say about what we had found and framed their reviews solely based on what they said were speculations about the significance of our work. We tried many journals (before this submission), and the outcomes were the same. You may ask: what was the main criticism?

 Simply put, reviewers did not like the hypothesis that the long tail meant tall train which would confer dominance on males which they could use in male-male competition and mate selection. We were forced down to remove almost any mention of male drive and male dominance in mate choice. And yet it is the same theory of male drive which was the basis of the mate choice theory of menopause which drew worldwide attention (Morton et al. 2013).

 In our opinion, Darwin missed out on the connection between the “mal-adaptive” long tail and the “dominance-conferring” tall train. A tall train allows males to compete against other males as well as serve as a big display to attract females. We thought we were proposing a new angle to look at the tail but it was treated as mere speculation. Evolutionary biology feeds on critical and thoughtful speculations as they become the basis for constructing new hypotheses. Darwin’s big book was all speculations, albeit very thought out and persuasive speculations. When a study is about testing a hypothesis the results and implications can be straightforward. Our study was not about testing a hypothesis; our study was about exploring the rules of pattern formation in the peacock tail and our findings had implications to mate choice, both males and females.      

 Our intention was more than simply to publish a paper. Our intention was to reopen the discussion on the female choice hypothesis which has dominated evolutionary biology for a century and a half and has shaped our thinking and societal sexual norms how mate choice works.

 We are pleased with the reviewer’s comments and with his Approval (with revisions) of the papers but also for brining tour attention to literatures that we had ignored, in particular, the paper on aposematic hypothesis and its application to the evolution of peacock’s train. We thank the reviewer for considering the publication of this paper and for enabling the discussion on the evolution of peacock’s tail. Reviewers’ comments are all very helpful and they will be taken into consideration in revising the manuscript.

 We sincerely thank the reviewer for suggesting these interesting papers. All the changes suggested by the reviewer have been made and all the references/works have been included and discussed. This has led to rewriting of the section “Implication for sexual selection theories and a hypothesis” in the Discussion and the conclusions in the Abstract.

 Rama Singh (Author)
